# *Aspergillus fumigatus*—Host Interactions Mediating Airway Wall Remodelling in Asthma

**DOI:** 10.3390/jof8020159

**Published:** 2022-02-06

**Authors:** Sara Namvar, Briony Labram, Jessica Rowley, Sarah Herrick

**Affiliations:** 1 School of Biological Sciences, Faculty of Biology, Medicine and Health, University of Manchester and Manchester Academic Health Science Centre, Manchester M13 9PT, UK; brionylabram@gmail.com (B.L.); j.rowley@imperial.ac.uk (J.R.); 2 School of Science, Engineering and Environment, University of Salford, Salford M5 4WT, UK

**Keywords:** *Aspergillus fumigatus*, asthma, airway remodelling, proteases, epithelial cells

## Abstract

Asthma is a chronic heterogeneous respiratory condition that is mainly associated with sensitivity to airborne agents such as pollen, dust mite products and fungi. Key pathological features include increased airway inflammation and airway wall remodelling. In particular, goblet cell hyperplasia, combined with excess mucus secretion, impairs clearance of the inhaled foreign material. Furthermore, structural changes such as subepithelial fibrosis and increased smooth muscle hypertrophy collectively contribute to deteriorating airway function and possibility of exacerbations. Current pharmacological therapies focused on airway wall remodelling are limited, and as such, are an area of unmet clinical need. Sensitisation to the fungus, *Aspergillus fumigatus,* is associated with enhanced asthma severity, bronchiectasis, and hospitalisation. How *Aspergillus fumigatus* may drive airway structural changes is unclear, although recent evidence points to a central role of the airway epithelium. This review provides an overview of the airway pathology in patients with asthma and fungal sensitisation, summarises proposed airway epithelial cell–fungal interactions and discusses the initiation of a tissue remodelling response. Related findings from in vivo animal models are included given the limited analysis of airway pathology in patients. Lastly, an important role for *Aspergillus fumigatus*-derived proteases in triggering a cascade of damage-repair events through upregulation of airway epithelial-derived factors is proposed.

## 1. Introduction

Asthma is a chronic respiratory condition that develops through a complex interplay of genetic and environmental influences, affecting at least 300 million people worldwide [1]. Symptoms include recurring episodes of persistent cough, wheeze and breathlessness, ultimately leading to poor quality of life [2]. Allergic asthma is characterised by sensitisation to environmental and biologic stimuli, such as pollen, dust mite products, animal dander, tobacco smoke and a wide range of fungi including *Aspergillus fumigatus (A. fumigatus)* [3,4,5]. Pathological features of asthma include airflow obstruction, due to mucus hypersecretion, and loss of cilia function, bronchial hyperresponsiveness, inflammation and airway remodelling [6]. Airway remodelling comprises of bronchial epithelial denudation, goblet cell hyperplasia and subepithelial fibrosis due to increased extracellular matrix (ECM) deposition [7]. Furthermore, remodelling involves increased smooth muscle mass, vascular remodelling and angiogenesis that correlates with deteriorating airway function [8,9,10]. Goblet cell hyperplasia causes excess mucus production which contributes to trapping foreign airborne particles but can also result in airway narrowing, whilst smooth muscle hypertrophy enhances the intensity of airway reactivity to such irritants. Much of our detailed knowledge with regards to airway remodelling comes from investigating the histopathology of patient bronchial biopsy samples, but these can be difficult to obtain and only provide a snapshot of the disease. High Resolution Computer Topography (HRCT), a useful non-invasive alternative for assessing airway remodelling, has demonstrated bronchial wall thickening and narrowing of the airway lumen in asthmatic lungs that correlates with airflow limitation [11,12,13,14]. Furthermore, HRCT scans have revealed the presence of bronchial obstruction caused by mucus-rich plugs, dilation of bronchi and co-existence of bronchiectasis, a widening of the airways associated with the thickening of the bronchial wall and presence of excess thick mucus [14,15]. In addition to contributing to asthma symptoms, airway remodelling may precede, or occur in parallel with inflammatory changes, making the development of targeted therapeutics to reduce these structural changes an important priority [9,16,17]. Despite this, pharmacological therapies mainly focus on inflammatory targets in asthma patients. Bronchial thermoplasty, an endoscopic procedure that delivers heat treatment to the airway wall to reduce smooth muscle mass, remains one of the few procedures that is proposed to modulate airway remodelling in uncontrolled asthma [18].

Inhalation of airborne components, including airborne conidia, hyphae and fungal fragments, is typically inconsequential for most but for some individuals, dependent on their individual immune status, it may cause a spectrum of disease ranging from sensitisation to severe invasive infection [19]. Intriguingly, the causative fungal species involved in disease may be geographically determined [20]. Furthermore, assessment of respiratory mycobiota in a UK population cohort showed that one of the dominant species present in the airway was *A. fumigatus* [21]. Another UK-based study found that the respiratory fungal load was much higher in severe asthmatics receiving corticosteroids compared with healthy controls, with *A. fumigatus* being one of the most prominent species [22]. *A. fumigatus* can grow both indoors and outdoors with an average adult inhaling several hundred conidia per day and, owing to their small size, they are able to reach the distal alveoli [23]. Following germination and subsequent growth, *A. fumigatus* produces a multitude of factors, some of which can act as allergens that may either mediate or aggravate asthma symptoms. Moreover, its ability to colonise the respiratory tract, if not readily cleared by inflammatory cells, means it may drive a sustained release of allergens and other products over a prolonged period of time [23]. However, despite strong evidence for an association between *A. fumigatus* and severe asthma, pathophysiological mechanisms driving an altered airway structure are still being elucidated. Our current understanding of *A. fumigatus*-associated asthma, fungal–airway epithelial cell interactions and proposed mechanisms leading to airway remodelling are discussed.

## 2. *Aspergillus fumigatus* Sensitised Asthma

In healthy individuals, inhaled conidia are removed by the innate immune system through phagocytosis, [24] whereas in immunocompromised patients, or those with reduced lung function, conidia clearance is less effective, allowing germination, prolonged host-allergen exposure and even fungal colonisation with possible invasion [19]. Global estimates indicate that approximately 28% of people with asthma are hypersensitive to *A. fumigatus* [25]. Furthermore, several studies have demonstrated that fungal sensitisation is associated with increased asthma severity, hospitalisation and even mortality [26,27,28]. A subtype of asthma, identified as ‘Severe Asthma with Fungal Sensitization (SAFS)’, collectively describes a diagnosis of severe uncontrolled asthma, serological or skin prick test detection of fungal sensitisation, and exclusion of Allergic Bronchopulmonary Aspergillosis (ABPA) [29]. In clinical practice, fungal sensitisation is detected by immediate skin hyperreactivity to fungal antigens and/or an increase in IgE antibodies (<1000 IU/mL) [30]. Interestingly, in a cohort of severe asthmatics, 19% showed bronchiectasis associated with poor lung function and frequent exacerbations which was commonly associated with *A. fumigatus* sensitisation specifically and positive sputum cultures [31]. Findings from another study indicated that 35% of severe asthma patients had central or peripheral bronchiectasis that was again highly associated with *A. fumigatus* sensitisation and/or sputum- positive cultures [32,33]. Moreover, bronchiectasis patients with persistent *Aspergillus* cultures were more likely to be hospitalised, have secondary infections, and a more severe lung function decline [34]. Whilst clearly a major health concern, there is limited information regarding histopathological changes of the airways and features of bronchiectasis in the lungs of SAFS patients. Another allergic hypersensitivity disease of the airways that complicates conditions such as asthma and cystic fibrosis and primarily associated with *A. fumigatus* is ABPA [19,29]. Compared with SAFS, ABPA presents with a more intense inflammatory response [29]. Clinical characteristics of ABPA include lung function deterioration, elevated *A. fumigatus* specific antibodies, higher than that observed in SAFS (IgE antibodies >1000 IU/mL) and airway eosinophilia [19]. The presence of highly thickened mucus is a hallmark of ABPA and is postulated to facilitate an uncontrolled vicious cycle of fungal growth, antigen production, inflammation and repair and worsening lung pathology [35]. Lung damage frequently localises to the proximal airway region, combined with granulomatous bronchiolitis as well as pulmonary fibrosis [29,36]. Similar to SAFS, bronchiectasis is also a common feature of ABPA, [37] although it remains unclear whether fungi cause or complicate established bronchiectasis, ultimately placing the patient on a pathway to ABPA development [38]. One study using HRCT reported comparable bronchial wall thickening, but a greater prevalence of bronchiectasis in ABPA patients compared with non-ABPA patients [39]. 

## 3. Interaction of Airway Epithelium with *A. fumigatus*

Airway epithelium, lying at the interface of the respiratory system and external environment, acts as one of the first lines of defence against inhaled conidia and hyphal fragments, and plays a central role in innate and adaptive immune responses (reviewed in [40]). Inhaled *A. fumigatus* conidia adhere to the airways in a number of ways including via exposed ECM components [41] and directly to airway epithelial cells [42], possibly by interaction with integrin receptors [43] and junctional complex protein, E-cadherin [44]. Conidia internalisation can lead to clearance by epithelial cells, but also inflammation and/or epithelial cell death (for review see [45]). It is conceivable that the exposed, damaged, mucus-rich environment of an asthmatic airway is an ideal platform for conidia entrapment, attachment, germination and growth. Interestingly, in cultures of airway epithelial cells, *A. fumigatus* conidia showed weak adherence to ciliated cells due to the action of cilia beat, but were able to adhere to non-ciliated cells [46]. In an organ culture model with human bronchial mucosal tissue, *A. fumigatus* conidia mainly adhered to mucus, microscopic indentations of damaged epithelial cells and, to a lesser extent, directly to epithelial cells [47]. FleA, a lectin expressed on the surface of *A. fumigatus* conidia, binds to purified mucin glycoproteins and so may facilitate this event [48]. Another favoured theory is that damage to and denuding of airway epithelium exposes basal lamina proteins supporting the adhesion of inhaled conidia [49]. Further support for this proposal comes from the fact that *A. fumigatus* conidia adhere to purified laminin and fibronectin as well as fibrinogen, types I and III collagen in culture [49,50,51,52,53]. Moreover, conidia bind to purified fibrinogen in a dose- and time-dependent fashion in vitro [54]. *A. fumigatus* also showed superior binding compared with less pathogenic species of *Aspergillus,* suggesting that adhesion to ECM may be important in disease pathogenesis [55]. Of note, *A. fumigatus* culture filtrate enhanced conidia–ECM adherence [49], suggesting that secreted fungal factors somehow facilitate binding to the damaged airway. 

In addition to acting as a physical barrier, airway epithelium recognises *A. fumigatus* conidia though expression of pattern recognition receptors (PRRs) such as C-type lectins and toll-like receptors (TLRs), that bind to pathogen associated molecular patterns (PAMPs) to elicit an immune reaction. The outer rodlet layer of inhaled *A. fumigatus* conidia renders them inert, but the shedding of this layer exposes immunogenic moieties [56]. The type of interaction between *A. fumigatus* and airway epithelium is dependent on the stage of germination. For instance, swollen or germinating conidia expose β-glucan residues on the surface that interact with the β-glucan receptor, Dectin-1. In addition, bronchial epithelial cells show increased expression of this receptor in response to *A. fumigatus* [57]. In vivo, loss of Dectin-1 receptor correlates with increased lung damage and cell death in response to *A. fumigatus*, suggesting that this receptor has a protective role against the effects of fungal exposure [58,59]. Gene silencing in human bronchial epithelial cells showed that Toll-like receptor 2 (TLR2) expression was essential for *A. fumigatus* induced Dectin-1 expression [57]. Based on observations made using immune cell cultures and animal models, it is likely that several other PAMP–PRR interactions, including those involving soluble receptors such as Pentadextrin 3, facilitate the epithelial response to *A. fumigatus* (for review see [60]). However, complete mechanistic studies are still needed to fully understand the detailed implications of these relationships. 

## 4. *A. fumigatus*-Induced Epithelial Cell Damage

The idea that the bronchial epithelium is dysfunctional in asthma, and that injury-induced repair events are activated is widely accepted (for review see [61]). Histopathological analysis revealed areas of denuded ciliated epithelium, as well as goblet cell hyperplasia, as common features of the asthmatic airway [10,62]. In addition, bronchiectasis biopsies showed extensive epithelial hyperplasia, with reduced lung progenitor cells, and an enhanced number of self-renewing cells capable of differentiation into ciliated and secretory cells [63]. Such observations may suggest that aberrant epithelial repair mechanisms contribute to the pathophysiology of asthma with associated bronchiectasis. 

As well as interacting directly with epithelial cells through PRRs, *A. fumigatus* also releases a host of soluble factors, such as allergensand secondary metabolites that can activate epithelial cells but also cause damage and loss of epithelial integrity [64]. The use of *A. fumigatus* culture filtrates, which are devoid of live spores or hyphae but include soluble secreted factors, are often used in laboratory studies to represent fungal exposure. Indeed, addition of culture filtrate from *A. fumigatus* to human nasal epithelial cells causes decreased transepithelial resistance, slowed cilial beat frequency and disrupted monolayer integrity [65,66,67,68]. Furthermore, cilial beat frequency reduction and epithelial cell detachment was observed in a human bronchial mucosa organ culture model exposed to germinating *A. fumigatus* [47,68]. Secondary metabolites, such as gliotoxin and verruculogen, are proposed to contribite to these effects [65,69]. However, *A. fumigatus* also produces a plethora of allergens, many of which have been cloned and produced as recombinant proteins [70] and more recently assessed as diagnostic tools in SAFS and ABPA [71,72]. Of note, many *A. fumigatus* allergens show proteolytic activity and include *Asp f* 5, a fungal matrix metalloproteinase (MMP), *Asp f* 10 a fungal aspartic protease and *Asp f* 13 and *Asp f* 18, fungal serine proteases [73]. Culture filtrates of *A. fumigatus* cause airway epithelial cell detachment in a dose-dependant fashion [74,75,76,77], and the addition of protease inhibitors, or the use of filtrates with diminished total protease activity, signficantly inhibited this effect [74,75,76]. Furthermore, both germinating *A. fumigatus* conidia and culture filtrate decreased cytoplasmic actin stress fibres, disrupted focal adhesions and induced membrane blebbing of airway epithelial cells. After 8 h, aggregates of polymerised actin formed in the cytoplasm and by 24 h, the actin cytoskeleton was almost completely depolymerised, the actin mesh disrupted and cell shrinkage and nuclear condensation observed [76,77]. These findings were significantly diminished in response to an *Asp f 13* mutant or in the presence of a serine protease inhibitor [76], suggesting that proteases have a major role in epithelial damage and the ensuing pathogical response to *A. fumigatus*. More recently, purified *Asp f 13* was shown to damage the integrity of bronchial epithelial cells grown at an air–liquid interface and the extracellular domains of junctional protein, E-cadherin [78]. The resultant junctional damage and mechanical force was shown to activate the mechanosensor TRPV4 and ultimatly drive airway inflammation and allergic sensitisation [78]. Our previous findings showed that *A. fumigatus* grown in complex protein growth media composed of homogenised pig lung or mucin resulted in filtrates rich in fungal serine protease and MMP activity, whereas low activity was found when cultured in minimal Vogels media [79]. It is envisaged that the fungus produces proteases to degrade complex proteins, thereby releasing factors required for growth. Indeed, proteases secreted by *A. fumigatus* have been found to degrade a range of ECM components [80,81,82]. Furthermore, we and others have demonstrated an important role for these fungal proteases in pro-inflammatory cytokine induction, including that of IL-6 and IL-8 [76,83,84,85], [85]. Indeed, when airway epithelial cells were cultured in the presence of germinating conidia from *A. fumigatus* strains previously shown to be low (Af293) or high (A1160) protease producers [79,85], secreted soluble factors were found to induce the pro-inflammatory cytokine response to strain A1160, which was diminished in the presence of MMP and cysteine protease inhibitors [85]. Taken together, the evidence suggests that *A. fumigatus* adheres to a range of epithelial and ECM components, activates PRRs, modulates epithelial function and ultimately drives inflammation. How these initial interactions between *A. fumigatus* and airway epithelium translate to longer-term airway remodelling events are less well understood. This is in part due to the difficulty in obtaining temporal histopathological airway biopsy samples from patients, but also technical issues associated with longer-term in vitro culture of *A.fumigatus* with epithelial cell for mechanistic studies. 

## 5. *A. fumigatus* Involvement in Driving Airway Structural Changes

**Figure 1:**
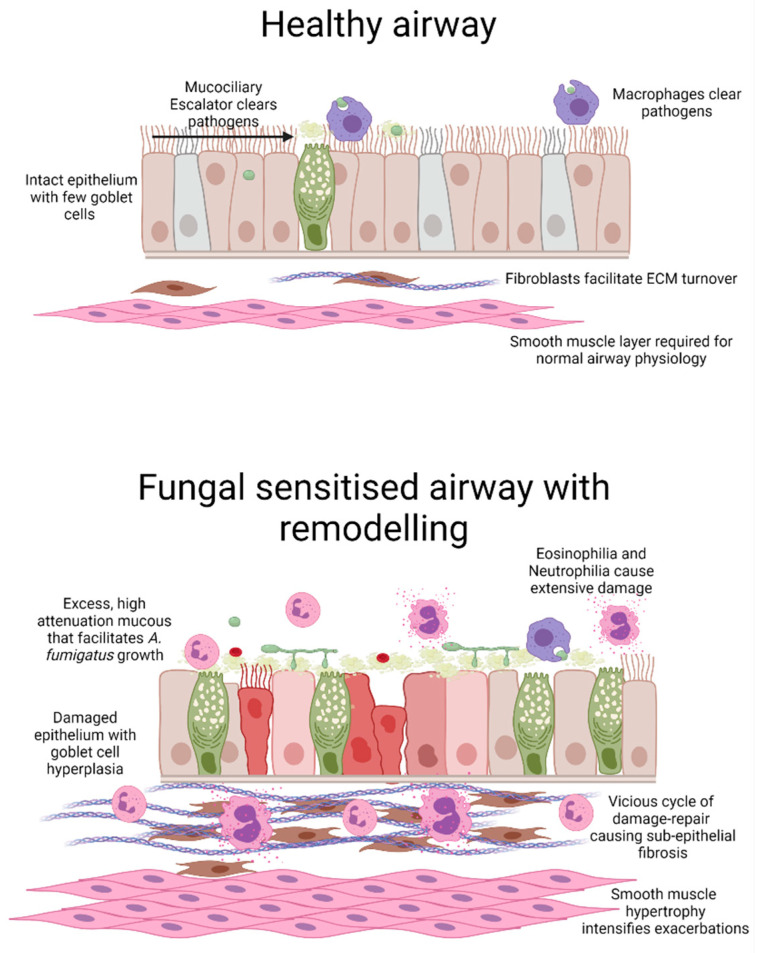
Schematic representation of *A. fumigatus*-induced airway wall remodelling. Compared with the healthy airway, a fungal-sensitised asthmatic airway displays extensive inflammation and remodelling. Airway remodelling includes areas of damaged epithelium coupled with goblet cell hyperplasia and excess mucus secretion which likely facilitates fungal growth. Coupled with increased inflammation, activation of fibroblasts and expansion of smooth muscle cells results in airway narrowing with possible luminal obstruction. Image generated in BioRender.

Although histopathological assessment of airway structural changes in lung biopsies from SAFS and ABPA patients is limited, consensus, mainly from CT scans, indicates that their airways show features of an asthmatic airway with extensive remodelling, subepithelial fibrosis and smooth muscle hypertrophy (Figure 1). Findings from murine models of *A. fumigatus* exposure provide a growing body of evidence to demonstrate that this fungus drives airway inflammation, remodelling and a deterioration of lung function (for review see [86,87]). In vivo models use a range of methods to deliver *A. fumigatus* to the airways, including conidia, extracts, purified antigens and culture filtrate via intranasal and intratracheal routes or by aerosolisation (Table 1) [88,89,90]. Porter et al. exposed mice to *A. fumigatus* conidia (unspecified strain) via an intranasal route, three times per week over the course of 18 days, resulting in airway hyperreactivity, eosinophilia and elevated IL-4 and suggested that conidia persist and drive inflammatory responses [90]. Another study exposed mice by aerosol challenge to conidia (strain B-5233/ATCC 13073, initially taken from pulmonary lesions [91]), twice a week for 4 weeks, causing peribronchiolar inflammation and evidence of Th2 sensitisation, subepithelial fibrosis and goblet cell hyperplasia [88]. Intriguingly, these pathological features were not present in mice exposed to melanin-deficient conidia (*∆alb1)*. Silver staining demonstrated that inhaled wild-type conidia germinated within the interstitium, whereas fewer *∆alb1* conidia remained and germinated [88]. Furthermore, Th2 and Th17 sensitisation was only apparent in mice receiving wild-type but not *∆alb1* conidia, demonstrating a crucial role for *A. fumigatus* melanin in germination and driving a host inflammatory process that contributes to airway pathology. 

Sustained colonisation of the airways with *A. fumigatus* conidia, similar to in patients with ABPA, is difficult to model in mice as the use of immunosuppressant agents in rodents can cause rapid invasive disease and mortality [87]. Urb et al. developed a novel solution by delivering *A. fumigatus* conidia (strain Af293, initially isolated from a neutropenic patient [91]) embedded in agar beads via an intratracheal route [89]. Exposure to conidia or agar beads alone caused no obvious signs of inflammation or airway pathology, whereas hyphal growth was visible in the airway lumen of mice exposed to agar beads with embedded conidia. Fungal growth was associated with significant inflammation, Th2 and Th17 sensitisation and evidence of airway remodelling, although structural changes were not quantified [87]. More recently, Jones et al. challenged mice intranasally with *A. fumigatus* conidia harvested from a patient with ABPA (strain W72310) seven times over the course of 2 weeks. Relative to mice being challenged with a more widely used strain (CEA10, derived from a patient with invasive aspergillosis [91]), a far greater number of W72310 conidia were found to persist in the lungs at the 14-day time point. This persistence was associated with relatively fewer infiltrating neutrophils and macrophages and a significant reduction in phagocytosis of conidia from the W72310 strain compared with the CEA10 strain [92]. In a separate experiment comparing W72310 strain exposed mice to vehicle control, the ABPA-derived isolate caused IgE induction associated with profound inflammation, goblet cell hyperplasia and increased lavage albumin [92]. Intriguingly, W72310 conidia that persisted in the lungs of mice and caused allergic sensitisation did so without evidence of extensive hyphal growth [92]. 

Several research groups have developed mouse models involving adjuvant *A. fumigatus* priming followed by intranasal conidia exposure, which may better model the impact of sustained allergen exposure. For instance, Hogaboam and colleagues gave mice commercially sourced intraperitoneal and subcutaneous injections of soluble *A. fumigatus* antigen, followed by a weekly intranasal antigen challenge for 3 weeks, and finally an intratracheal instillation of live conidia (strain 13073) [93]. This approach caused significant inflammation of the airways, Th2 sensitisation and airway hyperresponsiveness with many of the features apparent for up to 30 days post-exposure. Of relevance, histopathological analysis revealed significant subepithelial inflammation, goblet cell hyperplasia and subepithelial collagen deposition [93]. In a similar study, Hoselton and colleagues gave a single intraperitoneal and subcutaneous injection with commercially available *Aspergillus* antigen, followed by a series of intranasal challenges, completed with exposure to aerosolised conidia (unspecified ATCC strain) [94]. This regimen caused significant and even longer lasting Th2 sensitisation and airway hyperreactivity with, again, goblet cell metaplasia, subepithelial thickening with collagen deposition and smooth muscle hypertrophy evident at 35 days post-exposure [94]. To assess the role of B cells in airway remodelling, the same group compared inflammation and tissue pathology using two strains of mice—BALB/c mice that are particularly allergy prone and JH2/2 mice, which do not produce variable antibody heavy chain [95]. Comparable induction of subepithelial collagen deposition and airway hyperreactivity were observed, suggesting that B cells do not drive subepithelial fibrosis. However, mucus production was diminished, whilst eosinophilia, neutrophilia and some markers of Th2 and Th17 sensitisation were enhanced in JH2/2 mice compared with BALB/c mice [95]. These observations suggest disparate pathways may drive inflammation and the various features of airway remodelling in response to *A. fumigatus*. The role of Th2-inducing cytokines, IL-4 and IL-13, in driving airway pathology has also been assessed in murine models of *A. fumigatus* exposure. For instance, mice deficient in IL-4 repeatedly exposed to *A. fumigatus* antigen failed to develop an IgE response but showed comparable lung eosinophilia and lung pathology to that observed in wild-type mice [96]. These observations suggest that factors driving Th2 immunity and airway remodelling may in part be distinct. In another study of murine *A. fumigatus* antigen sensitisation, IL-13 neutralisation using specific antiserum caused a far greater reduction in airway hyperreactivity associated with a significant reduction in subepithelial fibrosis and goblet cell hyperplasia compared with controls or those receiving IL-4 antiserum [97]. These findings suggest that IL-13 plays an instrumental role in driving multiple features of airway pathology [97].

Taken together, animal models of *A. fumigatus*-induced airway inflammation demonstrate the ability of the fungus to persist in the airways of immunocompetent mice and drive pathological features, similar to that observed in SAFS and ABPA patients. Whilst these studies have been instrumental in developing a better understanding of disease evolution, the precise cellular mechanisms and the entire array of fungal components responsible remain to be fully described. As outlined in the subsequent section, evidence points to an important role for fungal-derived proteases in mediating *A. fumigatus*- induced airway wall remodelling.

**Table 1:** Findings from selected mouse models of *A. fumigatus* airway sensitisation.

Mouse Strain	*A. Fumigatus* Delivery Method	Key Findings	Ref
*A. fumigatus* conidia-only models			
C57BL6J	Intranasal delivery of 4 × 10^5^ conidia (isolated from household dust samples), three times per week over 18 days.	Relative to fixed conidia, live conidia caused airway hyperreactivity, eosinophilia, elevated IL-4 and IL-17 levels. Both live and fixed conidia caused modest neutrophilia.	[90]
BALB/c	Nose-only aerosol challenge with approximately 1 × 10^5^ conidia (strain B-5233/ATCC 13073) twice per week for 4 weeks.	Histology revealed persistence and germination of wild-type but not melanin-deficient conidia (*∆alb1)* 48 h post-final dose. Mice exposed to the wild-type but not *∆alb1* strain also displayed eosinophilia, neutrophilia Th2/Th17 sensitisation and evidence of subepithelial fibrosis and goblet cell hyperplasia by histology	[88]
C57BL6J	Intratracheal delivery of 2.5 × 10^6^ conidia (strain Af293) embedded in agar beads once.	Non-invasive fungal growth in airway lumen coupled with galactomannan detection. Robust inflammation, including Th2, Th17 and neutrophilia. Severe airway remodelling by histology	[89]
C57BL6J	Intranasal delivery of 1 × 10^7^ conidia (strain W72310 from ABPA patient or CEA10) seven times over 2 weeks.	W72310 but not CEA10 conidia persisted in the lung and could be detected as late as 28 days post-final exposure associated with eosinophilia, neutrophilia and Th2 sensitisation. Histology indicates subepithelial fibrosis and goblet cell hyperplasia in response to W72310.	[92]
C57BL6J	Intranasal delivery of 4 × 10^5^ conidia (strain Af293), three times per week over 18 days	Evidence of Th2 sensitisation, increased subepithelial fibrosis and epithelial thickening coupled with increased Endothelin-1 levels.	[98]
Systemic sensitisation followed by *A. fumigatus* conidia and/or antigen			
CBA/J	Systemic sensitisation by intraperitoneal and subcutaneous delivery of *A. fumigatus* antigen in Freund’s adjuvant, followed by a weekly challenge with *A. fumigatus* antigen for 3 weeks. In week 4, mice received an intratracheal dose of 5 × 10^6^ conidia (strain 13073).	Relative to conidia alone, pre-sensitisation caused profound Th2 sensitisation, profound eosinophilia, neutrophilia and peribronchiolar inflammation. Analysis of histopathology showed that pre-sensitisation also caused goblet cell hyperplasia and subepithelial fibrosis.	[93]
BALB/c	Systemic sensitisation by intraperitoneal and subcutaneous delivery of *A. fumigatus* antigen in alum. After 2 weeks, mice received a weekly intranasal dose of *A. fumigatus* antigen for 3 weeks and 1 week later, an estimated 6.6 × 10^5^ conidia (strain NIH 5233) were delivered by aerosol	Thickening of the epithelium, goblet cell hyperplasia and airway hyperreactivity persisted for at least 7 days post-final dose. Persistence of Th2 sensitisation and subepithelial fibrosis at the 35-day timepoint.	[94]
CBA/J	Systemic sensitisation by intraperitoneal and subcutaneous delivery of *A. fumigatus* antigen in Alum, followed by a weekly intranasal delivery of *A. fumigatus* antigen for 3 weeks. Finally, mice received a single intratracheal dose of 5 × 10^6^ conidia (strain not specified).	Upregulation of IL-4 and IL-13. Neutralisation of IL-13, but not IL-4 significantly reduced airway hyperresponsiveness, collagen deposition and subepithelial fibrosis as shown by histology.	[97]
BALB/c	Systemic intraperitoneal sensitisation with alum and crude *A. fumigatus* extract (strain not specified) followed by intranasal delivery of crude extract on days 25–27. In a separate group, mice received crude extract eleven times over the course of 5 weeks.	Alp1/Asp f 13 immunoreactivity visible in the submucosa of *A. fumigatus* sensitised mice.	[99]
*A. fumigatus* extract or culture filtrate models			
BALB/c and C57BL/6 derived genetically altered	Intranasal delivery of *A. fumigatus* extract (strain not specified), heat-inactivated extract or purified Alp1/Asp f 13, three times per week for 2 weeks.	Compared to mice receiving *A. fumigatus* extract or purified Alp1, those exposed to heat inactivated extract or Alp 1 showed diminished airway hyperreactivity, Th2 sensitisation, neutrophilia, peribronchiolar inflammation and goblet cell hyperplasia. Eosinophil-deficient and PAR2-deficient mice developed comparable inflammation, neutrophilia, Th2 sensitisation and goblet cell hyperplasia in response to Alp 1 to that found in wild-type mice.	[100]
BALB/c	Intranasal delivery of *A. fumigatus* sterilised and dialysed culture filtrate (CEA10 derived and protease allergen-deficient strains), twice per week for 4 weeks.	Neutrophilia, eosinophilia and Th2 sensitisation coupled with airway hyperreactivity and remodelling. Exposure to culture filtrates lacking protease allergens, Asp f 5 or Asp f 13, significantly reduced the extent of airway wall remodelling	[101]
C57BL6J	Intranasal delivery of *A. fumigatus* sterilised and dialysed culture filtrates (strain Af293), twice per week for 4 weeks.	Extensive inflammation and Th2 sensitisation in parallel with extensive subepithelial fibrosis. Endothelin-1 receptor antagonism prevented *A. fumigatus*-induced airway wall remodelling.	[98]

## 6. Involvement of *A. fumigatus*-Derived Proteases in Airway Wall Remodelling

Airway wall remodelling is a key feature of the fungal-sensitised asthmatic lung and demonstrated in vivo following prolonged *A. fumigatus* exposure (Table 1). Cellular and molecular mechanisms driving these structural changes are not fully understood and require further investigation, however, fungal-derived proteases produced during germination and growth are highly likely to be involved. Through using protease inhibitors and protease gene-deficient condidia, *A. fumigatus*-derived proteases have been shown to induce airway epithelial cell disruption with release of pro-inflammatory cytokines in addition to driving mucus production. Mucus hypersecretion may support the growth of inhaled conidia in the asthmatic lung and allow extended secretion of proteolytically active allergens resulting in a cycle of injury-repair with subsequent loss of epithelial integrity and long-term alterations in airway structure. Indeed, mucin-enriched culture media supported the production of *A. fumigatus* proteases [79] and such proteases have been found to upregulate mucin gene expression, *MUC5B* and *MUC5AC* [102], suggesting that a continual feedback loop may be in operation. Others have found that *Asp f 13* induced smooth muscle contraction in a murine precision-cut lung slice model [100] and upregulated production of host MMPs [99]. Interestingly, mice receiving intraperitoneal sensitisation followed by intranasal challenge with *A. fumigatus* antigen showed positive immunostaining for *Asp f 13* within bronchiole smooth muscle cells [103]. Such an observation mirrors clinical findings whereby *Asp f 13*, immunoreactivity was localised in the epithelium, mucus layer and smooth muscle of airway biopsies from asthmatic patients, in particular those patients with *A. fumigatus* sensitisation [99,103]. Our previous work demonstrated that *A. fumigatus* (derived from strain CEA10) produced high protease activity even in Vogels minimal media [101]. When mice were exposed intranasally to these protease-rich culture filtrates, they developed airway hyperreactivity, Th2 sensitisation and airway remodelling characterised by subepithelial fibrosis and goblet cell hyperplasia [101]. Of interest, exposure to culture filtrates derived from mutant *A. fumigatus* strains lacking *Asp f 5* or *Asp f 13*, only partially reduced airway inflammation, but significantly reduced features of airway remodelling [101]. Further evidence that fungal proteases drive structural changes comes from a related study, whereby mice were intranasally exposed to *A. fumigatus* extract or purified *Asp f 13* repeatedly over a two week period [100]. Both heat inactivation or the use of a serine protease inhibitor successfully prevented the deterioration of airway function, reduced lung lavage Th2 cytokines and infiltrating immune cells as well as goblet cell hyperplasia [100]. Intriguingly, *Asp f 13* directly elicited airway smooth muscle contraction, making targetted antagonism of fungal protease an attractive treatment option for *A. fumigatus*-sensitised asthma [100].

Endogenous host serine proteases, such as tryptase and thrombin, are known to activate lung epithelial cells through protease-activated receptors (PARs) via cleavage of the N terminus of the receptor [104,105]. PAR activation causes cytokine release as well as vasodilation, platelet aggregation, cellular proliferation and smooth muscle contraction [106,107,108]. PAR-2 is the most prominent PAR in allergic airway disease with increased expression on the surface of the epithelial cells of the bronchi in people with asthma [109]. Findings suggest that *A. fumigatus* proteases can also activate PAR-2 to drive inflammation and induce an increase in IgE as well as IL-6, IL-8, exotaxin, GM-CSF and MMP9 [110]. However, a role for *A. fumigatus*-derived proteases in activating PARs to mediate airway remodelling remains unclear. Suprisingly, compared with wild-type mice, PAR-2 knockout mice exposed to *Asp f 13* showed comparable airway hyperreactivty and goblet cell hyperplasia, and demonstrated a modest decrease in lung inflammatory cells and inflammatory cytokines [100], which may suggest that fungal proteases drive pathology through multiple mechanisms. In vitro, human airway epithelial cells showed an upregulation of mucin genes, *MUC5AC* and *MUC5B,* in response to *A. fumigatus* extract which was blocked in the presence of a serine protease inhibitor but not a PAR-2 antibody [111]. Rather, *A. fumigatus* proteolytic activity was shown to drive the phosphorylation/activation of Ras/Raf1/ERK to induce mucin production suggesting involvement of fungal proteases but independent of PAR activation [111]. Taken together, these observations indicate an important role for *A. fumigatus*-derived proteases in epithelial damage and aspects of airway remodelling which ultimatly leads to poor lung function.

## 7. *A. fumigatus*-Induced Airway Epithelial-Derived Profibrogenic Factor Production

**Figure 2:**
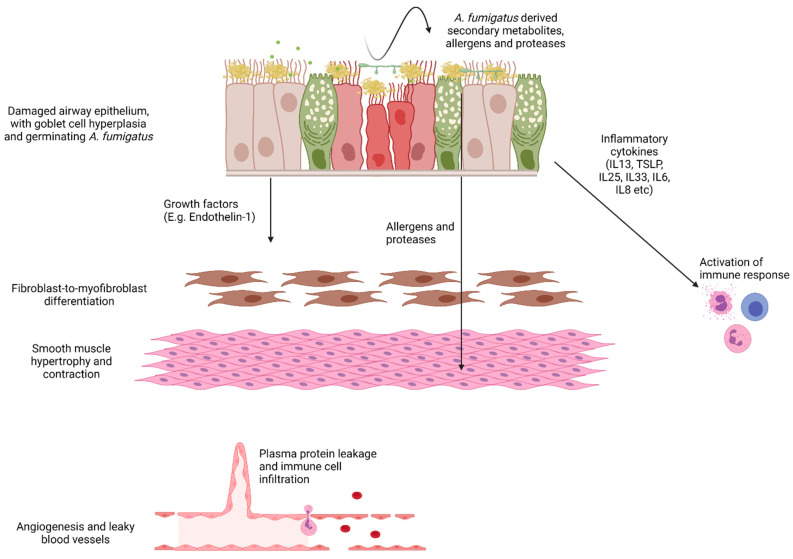
Schematic demonstrating possible pathways to *A. fumigatus*-induced airway remodelling. The asthmatic epithelium is characterised by goblet cell hyperplasia and mucus hypersecretion, loss of ciliated cells and subepithelial fibrosis. This fibrosis is likely driven by injury signals from the epithelium activating underlying fibroblasts. Loss of cilia function coupled with increased mucus and exposure of basement membrane components may enhance *A. fumigatus* adhesion and allergen production. *A. fumigatus*-derived factors drive the upregulation of pro-inflammatory cytokines to shape the immune response. In addition, epithelial-derived growth factors such as Endothelin-1 are upregulated in response to *A. fumigatus* and may directly activate underlying fibroblasts. Angiogenesis and vascular permeability support the arrival of infiltrating immune cells and circulating mediators likely contribute to airway wall remodelling. Image generated in BioRender.

It is speculated that cross-communication between airway epithelium and underlying mesenchymal cells, coined the ‘Epithelial-mesenchymal trophic unit (EMTU)’ shapes the way that the airway architecture changes in response to inhaled airborne agents [112]. Accordingly, the airway epithelium of asthmatic patients may respond to such exposure by secreting growth factors, which then drive an upregulation of ECM components [113]. In addition, evidence from genetically altered mice and inhibitor blocking studies suggest that these growth factors induce subepithelial fibrosis, which is linked to asthma severity [114,115,116,117,118,119]. In this regard, little is known about the ability of *A. fumigatus* to induce the production of fibrogenic growth factors by airway epithelial cells. Findings suggest that cultured murine lung epithelial cells exposed to both live and heat-inactivated *A. fumigatus* conidia induce an upregulation of epithelial-derived cytokines, such as thymic stromal lymphopoietin (TSLP), IL-25 and IL-33, that promote a Th2-type response, [120]. In a murine model of chronic multiple allergen exposure (house dust mite, ragweed, and *A. fumigatus* extract), treatment with anti-TSLP antibody reduced perivascular inflammation, reduced goblet cell hyperplasia and improved lung function [121] although remodelling of the airways was not analysed. Further studies investigating the role of TSLP, IL-25 and IL-33 in airway remodelling elicited by *A. fumigatus* are therefore required. Findings from another study showed that exposure to commercially sourced *A. fumigatus* antigens caused significant upregulation of peribronchiolar periostin, a matricellular protein, and features of airway remodelling, but mice deficient in periostin did not show any reduction of these features suggesting that periostin may not be a key mediator of airway remodelling [122]. We assessed the induction of key profibrogenic growth factors, TGFβ1, TGFβ2, periostin and Endothelin-1 by primary airway epithelial cells exposed to *A. fumigatus* conidia (strain Af293) or culture filtrate and found only a highly selective upregulation of Endothelin-1 [98]. Mice intranasally exposed to conidia or repeatedly with culture filtrate also displayed a highly selective upregulation of Endothelin-1. Furthermore, culture filtrate-exposed mice showed significant inflammation with Th2 sensitisation coupled with subepithelial fibrosis, whereas interestingly, antagonism of the Endothelin-1 receptor diminished this response [98]. How Endothelin-1 drives *A. fumigatus*-induced airway structural changes and the importance of Endothelin-1 in patients with *A. fumigatus* sensitised asthma still needs to be addressed (Figure 2).

Vascular leakiness is a key pathophysiological feature of asthma that correlates with induction of Vascular Endothelial Growth Factor (VEGF) and occurs in advance of inflammation in patients with deteriorating lung function [123,124]. Of importance, Endothelin-1 also promotes vascular permeability [125] and is upregulated in human asthmatic airways [126] as well as in an *A. fumigatus*-exposed airway mouse model [98]. Taken together, upregulation of VEGF and Endothelin-1 in the asthmatic airway may mediate vascular leak and facilitate the deposition of plasma proteins such as fibrin (Figure 2). Dysregulation of the fibrinolytic pathway in severe asthma patients treated with corticosteroid also favours fibrin deposition [127]. Indeed, the airway lumen of fatal asthmatics shows positive immunostaining for fibrin [128] and mucus plugs from airways of ABPA patients are rich in fibrin [129]. However, whether *A. fumigatus* interacts with fibrin to drive airway pathology remains to be elucidated. Of significance, co-administration of fibrinogen and thrombin were sufficient in driving airway disease in mice. The fibrinolytic enzyme, tissue plasminogen activator (tPA), also reduced airway reactivity [128], implying that coagulation and fibrinolytic pathways may contribute to airway remodelling and hyperreactivity. Of note, fibrin and Endothelin-1 are both known to mediate fibroblast activation and collagen deposition [130,131] supporting this concept.

In vitro, fibrinogen cleavage products are produced by incubation with proteases derived from another *Aspergillus* species, *A. oryzae* or thrombin (serine protease that converts fibrinogen to fibrin). These cleavage products can activate bone marrow-derived macrophages via TLR4 and support subsequent fungicidal activity to conidia, but may also contribute to the initiation or perpetuation of airway pathology [132]. It is plausible that *A. fumigatus* proteases may also cleave fibrinogen, producing by-products that activate PRRs. In another experiment, mice exposed to *Aspergillus oryzae* protease were compared to those receiving fibrinogen cleavage products. Results showed that cleavage products alone are not sufficient to drive airway disease to the extent observed with fungal protease. However, inhibition of thrombin in fungal protease exposed mice, attenuated all features of airway disease in this murine model, suggesting that the fibrinolytic pathway or degradation products play an important role in the mechanism of disease progression [132]. 

Taken together, fungal proteases may generate fibrin cleavage products in a similar manner to host proteases and these products may play a central role in driving both airway inflammation and airway remodelling. Linked to the concept that reduced fibrinolysis is involved in *A.fumgatus* airway effects, *Asp f 2*, a major *A. fumigatus* allergen and emerging biomarker for ABPA and SAFS [71,72], was recently shown to bind to plasminogen [133]. Plasmin, the active component of plasminogen, is a major fibrinolytic protease involved in fibrin matrix removal. In vitro, airway epithelial cells exposed to plasminogen-coated conidia in the presence of a plasminogen activator showed far greater epithelial damage than cells treated with conidia alone or conidia that lacked *Asp f 2* expression [133]. The actions of plasminogen-coated conidia were blocked using a serine protease inhibitor suggesting that an interplay between the major allergen *Asp f 2* and fibrinolytic protease activity is involved in inducing airway epithelial damage [133].

## 8. Conclusions

Taken together, the findings strongly suggest that the initial interaction between *A. fumigatus* and the airway epithelium is key to subsequent inflammation and damage-repair responses. In the asthmatic milieu, an environment rich in mucin, exposed matrix components and plasma proteins likely provides the ideal growth conditions for inhaled *A. fumigatus*. Through the course of germination, a wide range of allergens, some with protease activity, and metabolic by-products are produced which directly disrupt the integrity of the epithelium and elicit the productionof proinflammatory cytokines and fibrogenic growth factors. The ensuing recruitment of immune cells and further leakage of plasma proteins would support the development of a vicious cycle of inflammation, fibrin deposition and structural changes. *A. fumigatus* allergens with protease activity seem to be particularly important in disrupting the integrity of the epithelium, driving mucin production, subepithelial fibrosis, and hyperactivity of smooth muscle cells. Understanding the relative contribution of individual *A. fumigatus*-derived proteases in mediating these pathological events resulting in airway remodelling is now required. Future studies that quantify features of airway wall remodelling and the development of bronchiectasis in animal models would improve our knowledge of disease pathogenesis. Moreover, detailed analysis of the epithelium, subepithelial fibrosis and bronchiectasis in *A. fumigatus*-sensitised patients is also an important area for future investigation. As highlighted, a range of in vitro and in vivo models have been developed to investigate *A. fumigatus*- induced airway changes which have included using a wide variety of cell types (cell lines, primary cells, nasal, bronchial, alveolar), culture methods (submerged, air-liquid interface), different mouse strains, dosing regimens and fungal components (conidia/commercial extract/culture filtrate) from many *A. fumigatus* isolates. This variability in study design is likely to introduce heterogeneity in findings which should be considered, and a possible standardisation approach explored [91]. Lastly, although the current review has focused on the contributions of *A. fumigatus* to airway epithelial damage and subsequent pathology, it is recognised that a multitude of foreign agents are inhaled simultaneously, and the consequence of combined exposure is an area for further research. In particular, the important contribution of other fungi found to be associated with asthmashould not be overlooked. For instance, one study detected raised specific IgE levels against *Aspergillus*, *Candida* and *Trichophyton* in asthma patients, although some others such as *Cladosporium, Penicillium* and *Schizophyllum commune* were also observed [4]. In other patient cohorts, *Alternaria* and *Cladosporium* appeared to be especially frequent in patients with asthma and fungal sensitisation [5,134]. Detailed mechanistic studies investigating whether these other fungi and the factors they produce, can activate and damage the airway epithelium are limited [135,136]. Furthermore, emerging evidence points to an important role for sensation to a range of fungal allergens in the skin of atopic dermatitis patients, in particular those derived from *Alternaria* and *Malassezia*, *Aspergillus, Saccharomyces* and *Cladosporium* [135,137]. Such allergens may contribute to the process of ‘atopic march’, where sensitisation in the skin during infancy leads to subsequent development of allergic disease at other sites, including the airways. Indeed, higher frequencies of severe asthma and rhinitis are seen in atopic dermatitis patients with allergy to *Alternaria*, *Saccharomyces* and *Cladosporium* [137]. Taken together, fungal sensitisation is unlikely to be limited to one organism at a single body site and further large multi-centre global studies comparing the lung mycoflora and allergies, coupled with detailed in vitro and in vivo mechanistic studies, are required.
